# Assessment of In-Situ Gelling Microemulsion Systems upon Temperature and Dilution Condition for Corneal Delivery of Bevacizumab

**DOI:** 10.3390/pharmaceutics13020258

**Published:** 2021-02-13

**Authors:** Elena Peira, Giulia Chindamo, Daniela Chirio, Simona Sapino, Simonetta Oliaro-Bosso, Erica Rebba, Pavlo Ivanchenko, Marina Gallarate

**Affiliations:** 1 Department of Drug Science and Technology, University of Turin, 10125 Turin, Italy; giulia.chindamo@unito.it (G.C.); simona.sapino@unito.it (S.S.); simona.oliaro@unito.it (S.O.-B.); marina.gallarate@unito.it (M.G.); 2 Department of Chemistry and Interdepartmental Nanostructured Interfaces and Surfaces (NIS) Centre, University of Turin, 10125 Turin, Italy; erica.rebba@unito.it (E.R.); pavlo.ivanchenko@unito.it (P.I.)

**Keywords:** microemulsions, in situ gelling systems, corneal delivery, intraocular neovascularization

## Abstract

Bevacizumab (BVZ), a recombinant humanized monoclonal antibody, has recently been proposed as a topical application in the treatment of anterior segment neovascularization; however, as there are some disadvantages in the administration of common eye-drops, ophthalmic topical drug delivery systems are under study to improve the precorneal residence time, reducing the frequency of administration. In this work, oil-in-water and water-in-oil BVZ-loaded microemulsions are developed, able to increase their viscosity, either by the formation of a liquid-crystalline structure upon aqueous dilution, thanks to the presence of Epikuron^®^ 200 and polysorbate 80, or by body-temperature-induced jellification for the presence of Pluronic^®^ F127 aqueous solution as an external phase. In oil-in-water microemulsion, hydrophobic ion pairs of BVZ were also prepared, and their incorporation was determined by release studies. Microemulsions were characterized for rheological behavior, corneal opacity, in vitro corneal permeation, and adhesion properties. The studied microemulsions were able to incorporate BVZ (from 1.25 to 1.6 mg/mL), which maintained dose-dependent activity on retinal pigment epithelial ARPE-19 cell lines. BVZ loaded in microemulsions permeated the excised cornea easier (0.76–1.56% BVZ diffused, 4–20% BVZ accumulated) than BVZ commercial solution (0.4% BVZ diffused, 5% accumulated) and only a mild irritation effect on the excised cornea was observed. The good adhesion properties as well the increased viscosity after application, under conditions that mimic the corneal environment (from 1 × 10^3^ to more than 100 × 10^3^ mPa·s), might prolong precorneal residence time, proving these systems could be excellent topical BVZ release systems.

## 1. Introduction

Bevacizumab (BVZ) is a recombinant humanized monoclonal antibody, available in the EU as Avastin^®^ since 2005, directed against vascular endothelial growth factor (VEGF), which is responsible for the proliferation and migration of vascular endothelial cells causing tumor angiogenesis in vivo [1], which plays a central role in tumor growth, invasion and metastasis, also acting as a survival factor for endothelial cells via the inhibition of apoptosis.

BVZ is approved in the EU for the treatment of metastatic colorectal cancer, for the first-line treatments of metastatic breast cancer, advanced non-squamous non-small cell lung cancer, advanced renal cell carcinoma, advanced epithelial ovarian, fallopian tube and primary peritoneal cancer [2].

VEGF is also considered as the major angiogenic stimulus for intraocular neovascularization (NV), which is a severe complication of different inflammatory and ischemic ocular diseases [3] involving the anterior and posterior segment of the eye. Recently, the efficacy and safety of anti-VEGF agents (BVZ, ranibizumab, and aflibercept) in the diabetic macular edema treatment were assessed. Although other VEGF inhibitors are approved for the treatment of ophthalmological conditions, BVZ is currently used in ophthalmology and clinical practice all around the world [4].

BVZ was initially introduced as intravenous therapy for age-related macular degeneration (AMD); however, major disadvantages associated with systemic BVZ administration, such as the possibility of life-threatening adverse events [5], induced to use it off-label as intravitreal injection [6,7]. Clinicians worldwide continue to treat AMD with off-label BVZ, whose cost is a small fraction of that of ranibizumab (Lucentis^®^), and whose effectiveness appears to be analogous.

In recent years, many studies refer to the use of BVZ in the treatment of anterior segment NV, such as in corneal NV occurring because of inflammatory, traumatic, and metabolic diseases of the cornea itself and leading to a marked reduction in vision due to angiogenesis of abnormal vessels that block light [8]. Current therapies, based on the use of non-steroidal anti-inflammatory drugs (NSAID), steroids [9], prolactin, thalidomide, cyclosporine [10], angiostatin [11] and methotrexate [12] are not always completely successful. Although the results of recent experimental and clinical studies on the use of BVZ in both human and animal eye models suggest that it may be effective in the treatment of corneal and iris NV [13], the number of literature data, even if increasing, is still relatively small. Anyway, some studies [14,15] demonstrated the capability of topically administered BVZ to penetrate the neovascularized cornea without altering corneal transparency or exerting specific toxicity on both epithelium and endothelium.

Recent clinical studies [16] report on the safety and efficacy of topical applied BVZ in the treatment of corneal NV secondary to a variety of corneal diseases, such as glaucoma [17,18] and pterygium [19,20].

Although topical BVZ was well tolerated with no adverse events and no toxicity to the epithelium, keratocytes or endothelium was reported [14], in a clinical study conducted on seven patients [21], the duration and the dose of topical delivered BVZ seemed to be related with an increased risk of adverse reactions, as, by the second month of application, loss of epithelium integrity and stromal thinning were noted in 10 eyes.

That being said, the topical application of BVZ seems to be a non-invasive and practical procedure to treat several anterior segment diseases; however, there are some disadvantages that cannot be underestimated when common eye-drops are administered, such as the poor ocular bioavailability, due to high tear-fluid turnover rate and high nasolacrimal drainage. Novel ocular drug delivery approaches, including nanomicelles, nanoparticles [22], drug-eluting contact lenses, ocular inserts, ocular iontophoresis and ocular devices that prolong precorneal residence and enhance the bioavailability of the therapeutic agent are under study [23,24,25]. The primary goal of any ophthalmic topical drug delivery system is to improve the precorneal residence time, maintain a therapeutic drug concentration level at the target site, reduce the frequency of administration and overcome the various ocular barriers. Among the different nanosystems described in the literature, microemulsions (MEs), which are transparent colloidal oil-in-water (O/W) or water-in-oil (W/O) nanodispersions, that present sizes between 5 and 200 nm with significant thermodynamic stability and low surface tension [26], seem to be adequate to be administered topically as ophthalmic delivery systems owing to their ease of preparation and sterilization, and to their supersolvent properties allowing to deliver both hydrophilic and lipophilic drugs. Several studies on this topic are reported in the literature [27,28,29,30,31]. Recently, a comprehensive review paper was published underlying that MEs could significantly play a major role in ocular disease treatments as they are easily handled and cost-effective, but, up to today, applications found in the literature are still limited [32].

In recent years, increasing interest has grown to exploit systems able to thicken under external stimuli [33], such as temperature and/or pH changes or dilution-induced transformation from isotropic systems to liquid crystals. In situ-forming thermosensitive hydrogels are aqueous solutions, which jellify after a temperature change, owing to a temperature-induced phase transition governed by the balance of hydrophilic and hydrophobic moieties [34]. These systems, exhibiting low viscosity at 18 °C and forming gels at 32 °C, are generally based on natural polymers such as polysaccharides (cellulose and chitosan) or synthetic polymers (including PEG, poly-*N*-isopropylacrylamide, Pluronic^®^ F127) crosslinked by a variety of mechanisms [35,36].

Ideally, they can be easily administered topically as eye-drops, whose viscosity increases at body temperature, prolonging the retention time on the eye surface to provide and maintain an adequate drug concentration in the precorneal area.

The aim of the present paper is the development of BVZ-loaded MEs, able to prolong the residence time of BVZ on the corneal surface compared with conventional BVZ aqueous solutions and to promote BVZ corneal penetration.

To achieve this goal, several BVZ-loaded MEs are developed, using non-toxic and biocompatible ingredients, able to increase their viscosity, either by the formation of a liquid-crystalline structure upon dilution or by temperature-induced jellification of a thermosensitive polymer present in the continuous aqueous phase. Both MEs and the derived nanocomposite systems are characterized for physicochemical properties, corneal opacity, and in vitro corneal permeation. Moreover, in vitro cell viability assays on human retinal pigment epithelial ARPE-19 cells are performed to evaluate the effect of BVZ, after releasing from these systems, on cellular growth.

## 2. Materials and Methods

Avastin^®^ Roche (Basilea, Switzerland) was kindly purchased by Molinette Central Hospital (Turin, Italy). Deionized water was obtained by a Milli-Q system (Millipore, Bedford, MO, USA). Tween^®^ 20 (polysorbate 20), Tween^®^ 80 (polysorbate 80), hydroxyethyl cellulose, sodium phosphate monobasic and sodium phosphate dibasic were purchased from ACEF (Fiorenzuola d’Arda, Italy); trehalose dihydrate, cefuroxime, decyl polyglucoside, ethyl oleate, Pluronic^®^ F127, dioctyl sodium sulfosuccinate (AOT) from Merck (Darmstadt, Germany); Epikuron^®^ 200 (phosphatidylcholine 92%) from Cargill (Minneapolis, MN, USA); hyaluronic acid, sodium sulfate and sodium chloride from Alfa-Aesar (Ward Hill, MA, USA); Labrasol^®^ from Gattefossè (Saint-Priest, France).

Sulforhodamine B (SRB), dimethyl sulfoxide (DMSO), trichloroacetic acid, fetal calf serum and antibiotics for cell cultures were all purchase from Sigma-Aldrich (St. Louis, MO, USA). The retinal pigment epithelial ARPE-19 cell line (ATCC-CRL-2302) and the DMEM:F12 medium was purchase from ATCC^®^ (Manassas, VA, USA).

### 2.1. Corneas

Corneas were explanted from rabbits sourced from the slaughterhouse: eye-balls were excised within 2 h of the animal’s death, according to a protocol currently used for human cornea transplantation. They were transported to the laboratory chilled in normal saline (4 °C). A sclera ring of nearly 4 mm was maintained around the explanted corneas, which were kept in sterile Steinhardt medium for less than 1 week [37]. Before corneal opacity and permeation tests, corneas were assayed for their opacity by using the holder described below in the text, and corneas whose opacity exceeded 0.1 units of absorbance (λ = 570 nm) were discarded [38].

### 2.2. SEC-HPLC Analysis

The SEC-HPLC analysis method for BVZ quantification was performed as follows: BVZ solution from a freshly opened Avastin^®^ vial was diluted with normal saline (0.9% *w/w* NaCl) or with a solution, hereafter named “vehicle”, at the same composition of the commercial product (trehalose dihydrate, monobasic sodium phosphate, dibasic sodium phosphate, polysorbate 20). BVZ solution samples at increasing concentrations (from 5 to 50 µg/mL) were injected onto an SEC column, TSK-GEL G3000S Wxl 5 µm (300 mm × 7.8 mm, Merck KGaA, Darmstadt, Germany) for separation using a system comprised of a UV detector (Shimadzu SPD-10A at 280 nm, Shimadzu Corporation, Kyoto, Japan) and HPLC pump (Shimadzu LC-10 AD, Shimadzu Corporation, Kyoto, Japan) interfaced with a computer with Class VP Chromatography Manager Software (Shimadzu Corporation, Kyoto, Japan). The mobile phase consisted of 0.01 M pH 7.4 PBS added 0.1 mM Na_2_SO_4_. Linear regressions (R^2^ = 0.9995 and R^2^ = 0.9998) were obtained from the calibration curves determined using normal saline or vehicle solution, respectively, as dilution media. In release studies and permeation tests, SEC-HPLC analysis was conducted in triplicate for each sample for both batches.

### 2.3. AF4 Analysis

The analysis of the monomeric form of BVZ was performed using an AF2000 asymmetric flow field-flow fractionation (AF4) instrument (Postnova Analytics, Landsberg, Germany), combined with a UV-vis spectrophotometer operating at a wavelength of 280 nm. The channel was 350 μm thick, and regenerated cellulose membranes with 10 kDa cutoff were used. The flow rate of the outlet channel was set to 0.2 mL/min. The focusing step was performed for 4 min with a focusing flow rate of 2.3 mL/min. For the separation step, an initial constant crossflow rate of 4.0 mL/min for 35 min was set and then lowered to 0.00 mL/min in 10 min using a linear gradient. The mobile phase was: 9 mM phosphate buffer (pH 7.2) added with 137 mM NaCl (filtered through 0.1 mm Durapore membrane). The software NovaFFF AF2000 Control (Postnova Analytics, Landsberg, Germany) was used to set and control the flow rate values. Detection parameters and signal acquisition were controlled by NovaFFF Analysis.

### 2.4. Ion Pairs Preparation and Apparent Ethyl Oleate/Aqueous Solution Partition Coefficient Calculation

The ability of some counter ions to form hydrophobic ion pairs with BVZ increasing its lipophilicity, was studied. Bis(2-ethylhexyl) sulfosuccinate sodium salt (AOT) and cefuroxime (CEF) were chosen as counter ions. The ionic interactions were established between the residues of the basic amino acids of BVZ (ionizable amino groups) and the sulfonate group of AOT or the carboxylic group of CEF at pH 5.5. BVZ–AOT and BVZ–CEF ion pairs were prepared by mixing 80 µL of Avastin^®^ (containing 2 mg BVZ) and 2 mL of AOT or CEF solution (0.45 mg/mL) to obtain 1:150 molar ratio BVZ:counter ion. In the case of the BVZ–AOT, the 1:75 molar ratio was also studied. The obtained suspensions were centrifuged at 20,800 g (Eppendorf centrifuge, Hamburg, Germany). The supernatant was analyzed with SEC-HPLC and AF4, and the precipitated solid ion-pairs were dried under N_2_ flux. Apparent oil–water partition coefficient (P_app_) was determined using the shake-flask method. Each ion pair was dissolved in ethyl oleate and shaken at 22 ± 3 °C with an equal volume (1 mL) of 20% Pluronic^®^ F127 aqueous solution. The oil and the aqueous phase were then left to rest for 24 h until separation took place. Three replicates of each determination were carried out to assess reproducibility. BVZ molar concentration [BVZaq] was then determined in the aqueous phase by SEC-HPLC, and BVZ molar concentration in ethyl oleate [BVZoil] was obtained by the difference between the initial oil molar concentration [BVZ in] and [BVZaq]. From these data, Log P_app_ of BVZ ion pairs in ethyl oleate/polymer aqueous solution at pH 5.5 were determined, using the equation (1):

(1) $$\mathrm{Log}\;P_{\mathrm{app}}\;=\;\mathrm{Log}\;\frac{[BVZ_{in}]-[BVZ_{aq}]}{\left[BVZ_{aq}\right]}$$

Log P_app_ of the BVZ–AOT ion pairs (1:75 and 1:150 molar ratio) and of BVZ–CEF (1:150 molar ratio) were compared to Log P_app_ of free BVZ to confirm the formation of BVZ lipophilic ion pairs.

### 2.5. BVZ Stability

BVZ solutions (1.25 and 5 mg/mL) were prepared by diluting freshly opened Avastin^®^ with normal saline or with a vehicle. In order to detect any possible degradation or aggregation of the antibody in aqueous media, each BVZ solution, stored at 4 °C, was analyzed for monomeric BVZ content by SEC-HPLC over a 30 day period and compared with an identical BVZ solution obtained from freshly in the day opened Avastin^®^ vials to obtain decrease percentage (ΔC%) over time of monomeric BVZ, with the equation (2):

ΔC% = [(concentration of BVZ monomeric form in solution stored over time − concentration of BVZ monomeric form in freshly diluted Avastin^®^)/concentration of BVZ monomeric form in freshly diluted Avastin^®^] × 100 (2)

A 1.25 mg/mL BVZ solution in normal saline or in a vehicle, stored at 4 °C, and successively 1:10 *v/v* diluted in the mobile phase was also analyzed by AF4 to confirm the presence of monomeric peak over a 30 day period.

The secondary structural stability of Avastin^®^ diluted in normal saline, in a vehicle, in pH 7.2 phosphate buffer and in pH 10.5 carbonate buffer (0.1 M) were analyzed by circular dichroism (CD) spectroscopy. The analyses were performed with a circular dichroism spectropolarimeter (JASCO J-815, Jasco, Tokyo, Japan), equipped with a Xe arc lamp, to record data in the far-UV spectral range. CD spectra resulted from the average of 4 scans recorded for each sample at 100 nm/sec scanning rate, and they were acquired at 20 °C. The measurements were carried out using a quartz cuvette with a path length of 0.1 mm in the 190–240 nm wavelength range. All spectra recorded were corrected using the correspondent solvent medium as the baseline. Since the saturation signal (UV absorbance greater than 1.0) impairs the linearity of the response of the instrument in the CD signal, the measures required preliminary steps of dilution necessary to calibrate the concentration of BVZ in each sample. Data were analyzed by Spectra Analysis software, purchased by JASCO. The secondary structure content of BVZ in different aqueous solutions was predicted from UV-CD spectra by using K2D software. The stability of the BVZ–AOT (1:150) ion pair solubilized in 0.1 M carbonate buffer at pH 10.5 was also analyzed by the same technique to confirm the structural maintenance of BVZ once the ion pair breaks.

### 2.6. Formulative Studies of Microemulsion

In this study, ME-based phase transition systems at different viscosities were developed to propose them as ocular delivery systems for BVZ by topical instillation. The phase transition upon water dilution of a water-in-oil ME (W/O ME) system was studied. ME contained a mixture of Epikuron^®^ 200 and polysorbate 80 (1:1.1 *w/w*), as a surfactant, and Avastin^®^, diluted to 8.33 mg/mL of BVZ in a vehicle as aqueous phase, was prepared, and ethyl oleate was selected as oil phases, due to a fair solubilizing capacity of the resulting ME systems. The final BVZ concentration in the W/O ME formulation was 1.25 mg/mL. The phase transition of an oil-in-water ME (O/W ME) system upon temperature increase, from 18 ± 3 °C to 32 ± 3 °C, which is close to the internal temperature of the eye, was also studied. These systems comprised a mixture of Labrasol^®^ and polysorbate 80 (1:10 *w/w*) as a surfactant, ethyl oleate as oil phase, and Avastin^®^ diluted in the polymeric solution as the aqueous phase. The polymeric solution was composed of 20% Pluronic^®^ F127 in a vehicle. From approximately 32 °C upwards, an aqueous Pluronic^®^ F127 solution at high concentration (20% *w/w*) undergoes sol–gel transition. Therefore, the formulation is liquid at low-temperature, facilitating its application on the corneal surfaces and reverts to a viscous form at the temperature of 32 ± 3 °C. The final BVZ concentration in O/W ME was 1.6 mg/mL. BVZ was added in O/W ME free and as a hydrophobic ion pair to allow the incorporation of BVZ in the dispersed oil phase. In this case, the O/W ME was prepared to add all components to the ion pair previously precipitated. The presence of monomeric BVZ in MEs was confirmed by AF4 analysis.

### 2.7. Pseudo-Ternary Phase Diagram Construction

Pseudo-ternary phase diagrams were constructed using Origin software (Origin 8.0, OriginLab Corporation, Northampton, MA, USA). The pseudo-ternary phase diagrams of oil, surfactant mixture and aqueous phase were constructed at 25 °C using the water titration method to obtain the components and their concentration ranges that can result in a large existing area of the W/O ME. Surfactants were blended in fixed weight ratios (1:1.1). Aliquots of a surfactant mixture (TA) were then mixed with oil at room temperature. The oil:TA ratio varied from 5:95 to 95:5. Water or diluted Avastin^®^ in a vehicle was added dropwise to each mixture (oil:TA) under gentle magnetic stirring at room temperature. The resulting systems were then vortex mixed for 5 min and left to equilibrate overnight at room temperature. Following each addition of an aliquot of the aqueous phase, the mixtures were assessed visually. They were then characterized and classified based on their visual appearance (transparency) and behavior using polarized light to distinguish the liquid crystal region (LC) from the ME region. No heating was done during the preparation. The samples were marked as points in the phase diagram. The regions of existence of the ME and LC were demarcated. Amounts of all three phases were taken in *w/w* percentage. Based on this diagram, selected oil, surfactant, and co-surfactant were used for the preparation of the W/O ME that converts in the LC system by water dilution.

### 2.8. Rheological and Viscosity Measurements

Viscosity and rheological behavior of the W/O ME and LC were measured at 25 °C; O/W ME viscosity was measured at 18 and 32 °C using a Brookfield digital viscometer (DV-III+, Brookfield, Milwaukee, WI, USA) with SC4-25 spindle coupled to a temperature-controlling unit. The samples were thermostated at the required temperature with a circulating bath connected to the viscometer. Viscosity was recorded at a shear rate of 0.08 s^−1^. The rheological behavior was estimated by submitting the samples to increasing and then to progressively decreasing shear rates in an appropriate range (0.001 to 6 s^−1^) for each system. Rheograms were constructed by plotting shear stress as a function of shear rate.

### 2.9. Sol–Gel Transition Time of O/W ME

20% *w/w* Pluronic^®^ F127 aqueous solution shows interesting thermoreversible gelation behavior. This renders Pluronic^®^ F127 attractive in designing thermoreversible gels for many topical, injectable and controlled drug delivery. The sol–gel transition of the polymeric solution and of O/W ME was determined by a test tube inverting method [39] with temperature increments of 2 °C per step. Each sample was prepared in a 1 mL vial. After equilibration at 4 °C for 24 h, sample-containing vials were immersed in a water bath at a constant designated temperature for 15 min. The gelation temperature was characterized by the formation of a firm gel that remained intact when the tube was inverted by 180°.

### 2.10. DSC Measurements

DSC measurements were performed with a PerkinElmer (DSC7, PerkinElmer, Norwalk, CT, USA), equipped with an instrument controller Tac 7/DX (PerkinElmer) with elaboration software data Pyris Version 3.71, by heating the samples at 10 °C/min from 10 °C to 40 °C. Signals were recorded and used to localize the micellization phenomenon of Pluronic^®^ F127 in the external aqueous phase of O/W ME with and without BVZ compared with Pluronic^®^ F127 solution.

### 2.11. Release Studies of BVZ from O/W ME

To study the release of BVZ, free and as hydrophobic ion pair, from O/W ME, test tubes were filled with 1 mL ME, gently covered with 1 mL of pH 7.2 phosphate buffer (0.1 M) as receiving phase and placed in an incubator at 32 °C ± 0.5 °C [36]. The release of BVZ as an ion pair from O/W ME was also investigated at 18 °C ± 0.5 °C. At fixed times, the total volume of the receiving phase was withdrawn and analyzed by SEC–HPLC for BVZ determination. In the SEC-HPLC analysis of BVZ ion pairs, the receiving phases were diluted with 0.1 M carbonate buffer to break the ion pairs and to detect monomeric BVZ.

### 2.12. Corneal Opacity Tests

A corneal opacity test was performed adapting the Bovine Cornea Opacity/Permeability (BCOP) method, as reported by Casterton et al. [38], to evaluate the corneal toxicity of the systems. The opacity test was performed by clamping the cornea, through its scleral ring, in the appropriate holder and filling both the compartments with glutathione bicarbonate Ringer (GBR) buffer prepared according to the literature [40] as a control. Then, in the donor compartment, GBR buffer was substituted by the W/O and O/W ME formulations for 1 or 10 min. After withdrawing and washing for 15 min, the opacity was measured by determining the absorbance of the cornea (λ = 570 nm) clamped in the holder. The results were expressed as an increase of cornea absorbance (ΔAU), before and after incubation with the formulations under study. The holder was housed in a spectrophotometer (Lambda 2 UV-vis PerkinElmer, Norwalk, CT, USA) at λ = 570 nm for opacity readings with the help of suitable support so that the beam precisely crossed the donor, the receptor compartment and the cornea clamped in the holder.

### 2.13. Diffusion and Accumulation Studies in Rabbit Cornea

Muchtar et al. studied the diffusion of drugs through a rabbit cornea used as a membrane between two compartments [41]. In the present work, the experiments were carried out by using modified all-Plexiglas Franz diffusion cell on the W/O ME and LC containing free BVZ or O/W ME containing free BVZ compared to BVZ commercial solution. The holder used consisted of a Plexiglas structure, with a donor and a receiving compartment, respectively, on the epithelial and on the endothelial side of the cornea, which must be placed in the orifice, which divides the two compartments as reported by Gallarate et al. [30]. To minimize the irritation caused to the cornea by the holder itself, according to the literature [42], the Plexiglas structure O-ring clamped the scleral ring all around the corneal circumference; moreover, the holder structure allowed maintaining the natural cornea curvature. The area available for diffusion was 0.63 cm^2^. The receptor compartment was filled with 3 mL freshly prepared normal saline solution, constantly stirred at 150 rpm with a Teflon-coated magnetic stir bead and all air bubbles were expelled from the compartment. One mL of the formulated ME was placed on the excised cornea. The diffusion cell was kept in an incubator at 32 °C ± 0.5 °C. The permeation study was carried out for 24 h and samples were withdrawn from the receptor at the end of the experiments. The withdrawn samples were analyzed for drug content by SEC-HPLC. At the end of the 24 h experiment, the cornea was removed from the diffusion cell and washed three times with normal saline for 15 s. The cornea was cut into small pieces and extracted in 2 mL of normal saline for 4 h at 37 °C. The extract was analyzed by the SEC-HPLC method.

### 2.14. Adhesion Studies

To study the influence of ME on corneal adhesion, an empirical method was developed following the procedure described by Gallarate et al. [30]. A gel with the same surface tension of tear fluid (28 mN/m) was prepared; its composition was: 1% hydroxyethyl cellulose, 3% decyl polyglucoside, 5% hyaluronic acid, q.b. 100% water. The gel was spread on a 24 × 32 cm glass support and kept resting until hardening was reached. 0.1 mL of each sample was placed onto the shorter side of the hardened gel, and the support was sloped of 18°. The time needed to flow over a 10 cm-distance was evaluated for each preparation. The flow rate was calculated as distance/time (cm/s).

### 2.15. Cell Growth Assay

The BVZ activity after release from O/W ME was evaluated in retinal pigment epithelial ARPE-19 cells by using a sulforhodamine B colorimetric cell growth assay (SRB assay) modified by Vichai and Kirtikara [43]. ARPE-19 were routinely grown in DMEM:F12 medium, with the addition of 10% (*v/v*) fetal bovine serum, 1% (*v/v*) penicillin–streptomycin, and were maintained in standard conditions (37 °C, 5% CO_2_ and 95% humidity). For growth assay, BVZ released in phosphate buffer solution at a different time from O/W system was used. Briefly, ten thousand cells were seeded into 96-well plates. After 24 h of growth, cells were incubated for 48 h, in triplicate wells, with the samples properly diluted in the culture medium. Then, the cells are fixed on plates with 10% (*w/v*) trichloroacetic acid, washed and stained with an SRB solution (0.057% *w/v* in 1% *v/v* acetic acid) for 30 min. After washing with 1% (*v/v*) acetic acid and drying, the bound dye is solubilized with Tris-buffer (10 mM, pH 10.5), and the optical density (OD) was quantified photometrically (492 nm). The cell growth was expressed as a percentage (mean OD of treated cells/mean OD of control cells x 100). The experiment was replicated three times for all the incubation times.

### 2.16. Data Analysis

Data are shown as mean ± SD (standard deviation/number of replicate).

## 3. Results

### 3.1. BVZ Stability in Aqueous Solution

In Table 1, the concentration decrease percentage (ΔC%) over time of monomeric BVZ as Avastin^®^ diluted in normal saline and in a vehicle is reported, compared to freshly diluted Avastin^®^. Samples were analyzed by the SEC-HPLC method described in Materials and Methods.

As can be noted, the SEC-HPLC analysis showed that BVZ was more stable in a vehicle than in normal saline at both investigated concentrations.

**Figure 1:**
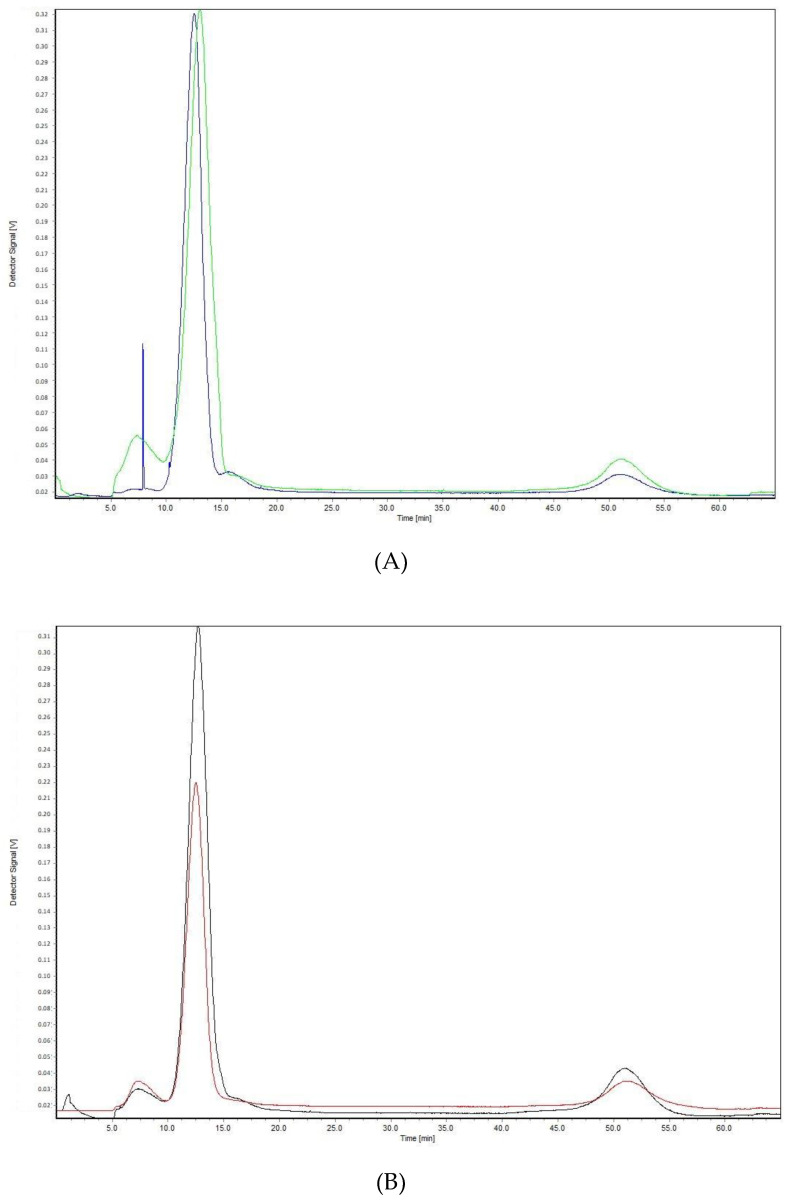
Fractograms of BVZ solution: (**A**) Avastin^®^ diluted in a vehicle at *t* = 0 day (green) and at *t* = 30 days (blue); (**B**) Avastin^®^ diluted in normal saline at *t* = 0 day (black) and at *t* = 30 days (red).

The same results were confirmed by analyzing 1.25 mg/mL BVZ aqueous solutions (in normal saline and in a vehicle) by AF4 analysis, as shown in Figure 1. These analyses were performed with the crossflow at 4 mL/min that allows a sharper peak of BVZ compared to that obtained with lower cross flows (2.0 and 0.5 mL/min).

**Figure 2:**
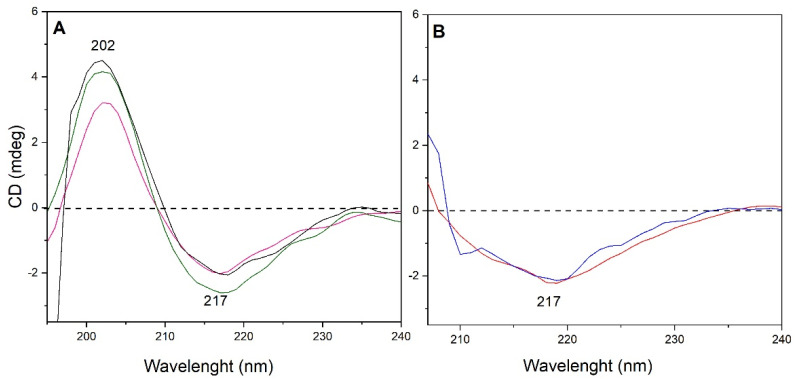
CD-UV spectra of BVZ (**A**) in a vehicle (green curve), normal saline (black curve), and phosphate buffer (magenta curve) and of BVZ (**B**) in the form of an ion pair (red curve) and after a break of ion pair (blue curve).

By CD analysis, BVZ secondary structure was maintained in aqueous solution (vehicle, normal saline, phosphate buffer, see Figure 2A, green, black and magenta line, in the order) after 24 h also when BVZ was present as an ion pair (BVZ:AOT 1:150) and when this ion pair was broken in pH 10.5 carbonate buffer (see Figure 2B, red and blue lines, respectively). All the spectra of BVZ in aqueous solutions have a similar shape, exhibiting a negative band with a minimum at 217 nm followed by a maximum at 202 nm. A similar profile is reported to be characteristic of the β-sheet type of structure [44].

Further, the analysis of the spectra of BVZ as ion pair and when it was broken with the carbonate buffer (Figure 2B) is hindered by the presence of electrolytes rendering the sample nontransparent below 205 nm (see corresponding CD Absorbance spectra in Figure S1). However, the visible part of the spectra exhibits a shape similar to those of BVZ in aqueous solutions (Figure 2A), meaning the well-defined minimum at 217 nm and the similar shape of the “slope” between 220 and 240 nm. In addition, it is worth mentioning that the fitting of these spectra with the same algorithm indicated a similar number of β-sheet structures (ca. 43%).

### 3.2. BVZ Ion Pair Formation and Log P_app_

Hydrophobic ion pairing of BVZ with AOT or CEF improved the lipophilicity of BVZ [45]. In Table 2, the yields (expressed as a percentage) of ion-pair formation and Log P_app_ values (±SD) in ethyl oleate/20% *w/w* Pluronic^®^ F127 aqueous solution at pH 5.5 are reported. The yield was calculated by determining BVZ concentration in the supernatant, after ion pair precipitation, by SEC-HPLC analysis. The presence of BVZ in monomeric form was confirmed by AF4 (data not shown).

The formation of the ion pair with AOT increases Log P_app_ of BVZ almost thrice. As CEF has many hydrophilic groups in its structure, the BVZ–CEF ion pair resulted less lipophilic than the BVZ–AOT one. As AOT has surfactant properties, at the higher concentration employed (BVZ:AOT 1:150 molar ratio), it can form micelles able to solubilize the BVZ phenomenon, which could explain the decrease of ion-pair oil partition at AOT concentrations higher than CMC. In fact, 1:75 BVZ:AOT showed greater lipophilicity than 1:150 BVZ:AOT.

### 3.3. Water-in-Oil ME Formulation Studies

**Figure 3:**
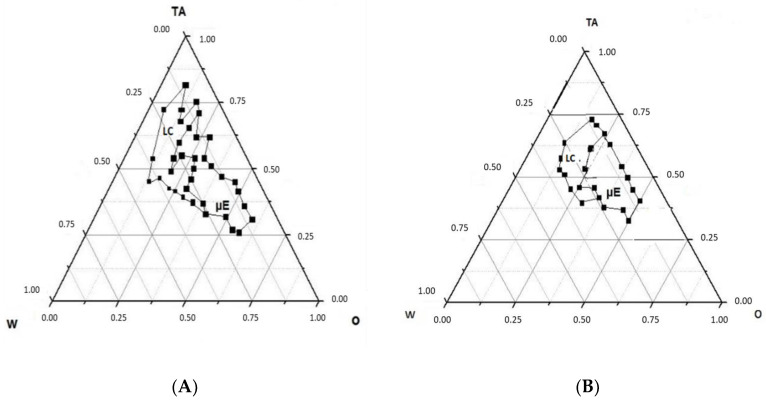
Pseudo-ternary phase diagram of the microemulsion (ME)–liquid crystal region (LC) system (**A**) without BVZ; (**B**) with BVZ.

The field of existence of the W/O ME systems that could present a change in viscosity by simple dilution by switching to a liquid crystal structure was studied. The pseudo-ternary phase diagram of the W/O system (Figure 3A) showed two distinct regions with the W/O ME transforming to lamellar liquid crystalline structure (LC) with increasing water content. The pseudo-ternary phase diagrams for blank ME regions were constructed using ethyl oleate as an oil (O), polysorbate 80 and lecithin mixture as a surfactant (TA) and water as the aqueous phase. For ME/LC regions containing BVZ, diluted Avastin^®^ (8.33 mg/mL of BVZ) was used as an aqueous phase (Figure 3B). Each point was evaluated by using a couple of polarized lenses. We remarked that in the presence of BVZ, both the fields of the existence of LC and ME are reduced.

### 3.4. Preparation of Microemulsion

After the identification of the ME region in the pseudo-ternary phase diagram, W/O ME formulations were selected at the desired component ratios to switch from the ME system to LC by dilution, miming the corneal environment. The preparation of selected W/O ME was performed in an easy way by putting the weighed components in a vial and stirring to form a clear ME. According to the pseudo ternary phase diagrams (Figure 3), liquid crystal systems were also prepared and observed using a couple of polarized lenses.

O/W ME (named PL1) was prepared by adding a 20% *w/w* Pluronic^®^ F127 aqueous solution in oil and surfactants mixture. Oil, surfactant blend and aqueous phase were vortex-mixed for 10 min, and the resulting systems were left to equilibrate overnight at room temperature before conducting the characterization tests. Table 3 reports the composition of the tested formulations with and without BVZ. These formulations included W/O ME and LC formulations (named M1 and LC1) containing aqueous phase at 16% and 30% (*w/w*), respectively, with the latter being close to the W/O ME–LC phase boundary. BVZ was incorporated by replacing water (M2 and LC2)or Pluronic^®^ F127 aqueous solution (PL2)with an Avastin^®^ dilution. In the case of O/W ME containing BVZ/counter ion, the ion pair was added in the oil phase before ME preparation.

The presence of monomeric BVZ in the ME systems was confirmed by AF4 analysis. It is possible to distinguish the peak of monomeric BVZ still remaining in the disperse system (Figure S2, Supplementary Materials).

### 3.5. Rheological Characterization and Viscosity Measurements

**Figure 4:**
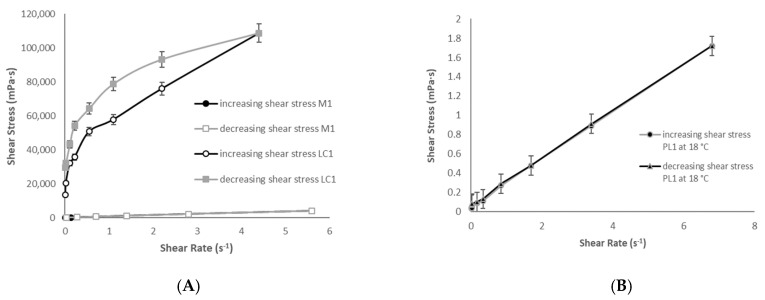
Rheological behavior of the W/O ME (M1) and LC system (LC1) at 25 °C (**A**) and of O/W ME (PL1) at 18 °C (**B**).

The rheological behavior at 25 °C of the W/O systems was studied. The conversion of Newtonian ME to non-Newtonian LC upon aqueous phase addition is shown in Figure 4A. In the M1 rheogram, the up curve is coincident with the down curve, showing time-independent viscosity, while the LC1 rheogram shows a thixotropic behavior. In Figure 4B, the rheological profile of PL1 at 18 °C is reported. It was not possible to characterize the rheological properties of PL1 at a temperature higher than that of gelation of Pluronic^®^ F127 (32 °C), while the rotation of the spindle determined cavitation phenomena in the system, which hindered the correct measurements of shear stress.

The viscosity values of the studied systems obtained at 0.08 s^−1^ shear rate are reported in Table 4, and the PL1 viscosity as compared to that of 20% Pluronic^®^F127 solution at the same temperatures.

The increase in viscosity of the W/O system is remarkable when M1 converts to LC1. Therefore, a highly viscous LC system, formed upon dilution of the M1 by the tear film, may remain on the cornea for a long time.

In PL1, being a thermosensitive system, thanks to the presence of Pluronic^®^ F127, a significant viscosity increase was noted upon temperature enhancement (from 18 °C to 32 °C).

Pluronic^®^ F127, able to form a thermoreversible gel in aqueous solution at 20% *w/w* (with gelation time = 1 min), maintained its ability in the ME formulations around body temperature, with a gelation time of 3 min when BVZ was in the external phase and of 1 min, 47 s when BVZ:AOT (1:150) ion pair was in the inner oil phase.

### 3.6. DSC Analysis

**Figure 5:**
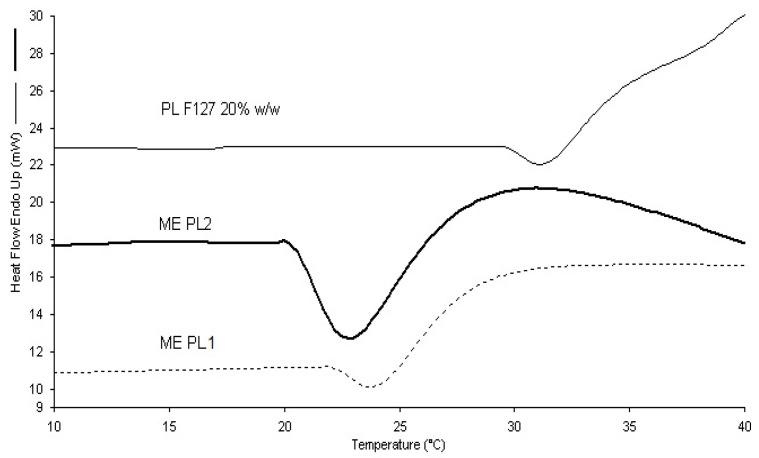
Thermograms of PL with (PL2) and without (PL1) BVZ, compared to thermogram of Pluronic^®^ F127 solution.

The possible impact of oil droplet addition upon Pluronic^®^ F127 gelation must be investigated. The endothermic micellization process of Pluronic^®^ F127 is usually analyzed by DSC. In this work, the possible impact of oil droplet addition on Pluronic^®^ F127 micellization was investigated (Figure 5).

Heat flow measurements performed during heating ramps were scrutinized in order to evaluate the possible loss of the thermal behavior related to micelle formation of Pluronic^®^ F127 in the presence of oil droplets. The large endothermic peak was ascribed to the micellization of Pluronic^®^ F127 unimers [46]. During heating, the rupture of hydrogen bonds of poly-(propylene oxide) (PPO) leads to an increase of hydrophobicity, while the poly-(ethylene oxide) (PEO) chains remain hydrophilic. Then, when the micellization is achieved, Pluronic^®^ F127 micelles arrange themselves in a crystalline structure [47] at a distinct temperature. Gelling temperature coincided with the crystallization temperature, which is difficult to study by DSC because slightly endothermic. The temperature-dependent micellization behavior of Pluronic^®^ F127 solution was maintained in PL1 and PL2 formulation. The micellization capacity of Pluronic^®^ F127 in PL2 about 22 °C probably allows retaining BVZ to a greater extent, even if in the external phase of the ME.

### 3.7. Release of BVZ from PL2

In order to investigate the ion-pair formation influence on BVZ release from PL2, diffusion tests were performed. The percentage of released BVZ over time from PL2 compared to Pluronic^®^ F127 solution at 32 °C is reported in Table 5. BVZ was released quickly and in a similar way (about 90% in 24 h) when incorporated in the polymeric gel or in PL2, as it is located in the external phase of the ME, where it is probably micellized into Pluronic^®^ F127 chains as well as in Pluronic^®^ F127 reference solution. When incorporated in PL2 as hydrophobic ion pairs, BVZ was released very slowly, almost 15–25% over 24 h. This behavior suggests that BVZ, in this case, is located in the inner oil phase of the ME. The release of BVZ hydrophobic ion pairs from PL2 was also tested at 18 °C. As at this temperature, PL2 viscosity is lower than at 32 °C, the release of BVZ was faster than from the viscous system.

Comparing BVZ–AOT and BVZ–CEF ion pairs, the higher the value of Log P_app_, the lower the release rate. Indeed, release results confirmed that the BVZ-CEF ion pair produced a faster release than the BVZ–AOT one, being less lipophilic. According to these results, 1:75 BVZ–AOT ion pair was not tested in release studies as the high value of Log P_app_ would probably produce a BVZ release too low for our purposes.

### 3.8. Corneal Opacity Tests

Irritant-induced opacity, which is experimentally determined by the amount of light transmission through the cornea, is an indicator of protein denaturation, swelling, vacuolization or damage in the epithelial and/or stromal layers. The evaluation of common standard irritants provides an empirical irritation scale, as reported by Battaglia et al. [48]. In this irritation classification, the samples result mild when opacity is ˂0.400 at λ = 570 nm. In Table 6, the increase of cornea absorbance (ΔAU) after contact with BVZ-containing ME was reported. After 1 min and 10 min exposition, a mild irritation effect was noted for all the systems under study. GBR buffer was used as a control.

### 3.9. Permeation and Accumulation of BVZ Through Rabbit Cornea

In Table 7, the percentage of BVZ diffused through the cornea and the percentage of BVZ accumulated in 24 h deposition are reported.

Increasing the water content of the delivery system (from M2 to PL2), an increase in BVZ corneal permeation was noted. BVZ released by the liquid crystal system (LC2) accumulates on the cornea more than the other systems under study.

### 3.10. Adhesion Properties of W/O ME

To evaluate the adhesion properties of the M2 and LC2 formulation compared to a conventional solution (distilled water), the course of the systems on the hardened gel was timed. The flow rates (cm/s) of M2 and LC2 are reported in Table 8.

PL2 was not tested because it was difficult to perform the study at 32 °C. The developed systems have a good adhesive capacity compared to water; therefore, they are probably able to remain on the cornea longer than an eye drop. In particular, the liquid crystal system is characterized by a high adhesive capacity, as it was not able to cross the gel, stopping halfway.

### 3.11. Effect of BVZ Released from O/W Systems on Cell Growth (Cell Growth Assay)

**Figure 6:**
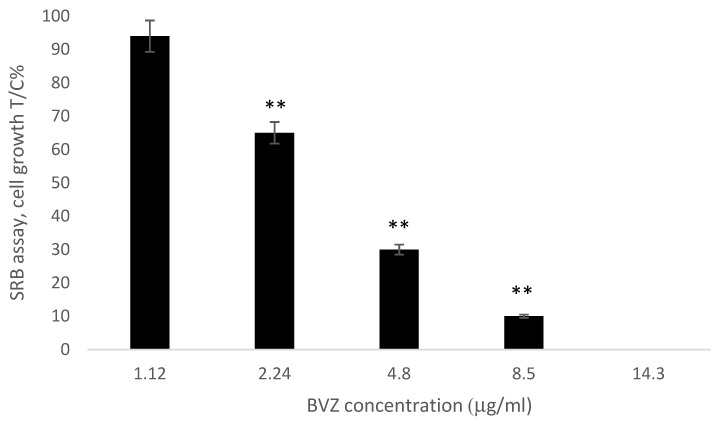
Effect of BVZ on ARPE-19 cell proliferation after 48 h of incubation. Cell growth is expressed as% T/C (mean optical density (OD) of treated cells/mean OD of control cells × 100). Values are mean ± SD (n = 3 wells/condition) of three independent experiments (** *p* < 0.01).

The cell growth assay was performed on ARPE-19 cells. This cell line was employed in this study as a model of retinal pigment epithelium (RPE) cells, able to secrete the VEGF [49]. The effect on cell growth of BVZ released from O/W ME (PL2) at the different times was tested at different concentrations determined as reported in the release studies. Cells were incubated for 48 h with each released sample diluted 1:100, and cell growth was determined by SRB assay (Figure 6).

As shown in Figure 6, a significant decrease of ARPE-19 cell viability after 48 h of incubation was observed for BVZ at concentrations higher than 2.24 μg/m (** *p* < 0.01). The effect of BVZ released from O/W ME was similar to the viability inhibition of BVZ commercial solution observed in a previous work [50]: at 5 μg/mL, the cell growth was decreased by 75%.

## 4. Discussion

The main goal of the present work was to develop BVZ-loaded MEs to be proposed as ophthalmic local delivery systems to promote BVZ corneal permeation and/or accumulation. MEs are thermodynamically stable disperse systems that are quite interesting for their supersolvent properties due both to the presence of oil and an aqueous phase and of a complex surfactant/cosurfactant mixture. These characteristics, together with the ability of the W/O microemulsions to evolve in liquid crystals upon water dilution, offer a plethora of possible drug carrying and drug release modalities, which are non-easily obtainable with other nanosystems.

Preliminary studies on BVZ stability were performed to evaluate in which aqueous solution was more appropriate to dilute Avastin^®^. Indeed, physicochemical instability of the antibody, which can lead to denaturation and/or aggregation, must be avoided; for this reason, it was necessary to assess the stability of the monomeric form of BVZ under the conditions of use. The mixture of polysorbate 20, trehalose and phosphate salts in water was the most suitable for dilution while maintaining the stability of the monomeric BVZ, probably owing to the presence of polysorbate 20, which formed micelles able to stabilize BVZ. This hypothesis seems to be also confirmed by the stability of BVZ in normal saline, which was significantly lower at 1.25 mg/mL than at 5 mg/mL concentration: as 1.25 mg/mL solution was prepared to dilute a smaller volume of Avastin^®^ than that used to prepare 5 mg/mL solution, polysorbate 20 (present in Avastin^®^) was diluted below its CMC value, determining consequent instability phenomena of BVZ. BVZ maintained its structure also when assembled in the ion pair, as shown with CD spectra. The fitting of the CD spectra (Figure 2) reveals the high relative amount of b-strand (ca. 45%), which indeed corresponds with the detailed analysis of the secondary structure of BVZ carried out by Ferreira et al. [51] and confirms the relative abundance of the β-sheets (ca. 47%).

These results are strongly supported by those obtained using three different analytical techniques (i.e., SEC-HPLC, AF4 Analysis, CD), which, though not interchangeable, can supply complementary information to assess BVZ moiety integrity after the production technologies reported in the present research. This was an important and absolutely nonobvious prerequisite to developing BVZ-containing microemulsions.In this research, O/W and W/O MEs were proposed for their in situ gel-forming properties and potential utilization for ocular drug delivery to reduce the frequency of instillations per day. Thanks to the construction of the pseudo-ternary phase diagram, it was possible to evaluate the appropriate dilution to obtain the corresponding LC system from the W/O ME.

A W/O ME was developed that was able to incorporate BVZ in the aqueous inner phase and to increase its viscosity once in contact with an aqueous environment, owing to the formation of liquid-crystalline structures in the system.

The field of existence of the W/O ME and its switching into an LC structure upon water dilution was investigated by pseudo-ternary phase diagrams systems. LC might form when W/O ME comes in contact with the physiological tear fluid and might influence both drug release and precorneal residence time. The reduced existence area of the W/O ME in the presence of BVZ can be related to a probable interaction of BVZ (even if located in the inner phase) with the W/O interface, perturbing, although not dramatically, the phase assessment. Diluting with Avastin^®^ solution, of course, determined a variation of BVZ concentration dependent on added Avastin^®^ volume, giving some supplementary information about the maximum amount of BVZ that it was possible to introduce in the studied W/O ME, but this was not the goal of the present research.

Furthermore, an O/W ME was proposed, containing a solution of Pluronic^®^ F127, a polymer characterized by a sol–gel transition around 32 °C. This system has proven to be able to incorporate a commercial solution of BVZ, maintaining the temperature-sensitive characteristics of the polymer solution. BVZ maintained the monomeric form when incorporated in microemulsions, as shown with AF4 analysis (Supplementary Materials). As in the O/W microemulsion system, BVZ is probably located in the external phase, hydrophobic ion pairs of BVZ were also prepared. Hydrophobic ion pairing of BVZ with AOT or CEF proved to improve the lipophilicity of BVZ, necessary to incorporate it in the inner phase of O/W ME. Moreover, as CEF is one of the widely used antibiotics to prevent endophthalmitis, which might also occur during invasive procedures such as intravitreal injections or cataract surgery, we hypothesized that BVZ–CEF ion pair might play a double therapeutic action. On the other hand, AOT is a surfactant already widely studied as a counter ion of BVZ, which forms a highly water-insoluble ion pair [45]. Even if the use of the term lipophilicity can sound as not properly used, as no octanol/water partition was determined, it indicates the increase of ethyl oleate/water partition, as ethyl oleate is the oil phase of the ME, in, which we aimed to dissolve a quite hydrophilic molecule like BVZ.

The very low viscosity often exhibited by MEs is inappropriate for ophthalmic use, as they may easily be removed from the eye surface by lacrimation and/or nasolacrimal drainage. In the present research, ME’s viscosity was enhanced by exploiting two different strategies: the addition of water to the W/O ME to turn it into a liquid crystal system and the introduction of a thermogelling polymer such as Pluronic ^®^F127 in the aqueous phase of O/W ME.

Release studies performed on the different prepared systems allow us to deduce a number of conclusions.

First of all, BVZ release studies from PL2 confirmed the incorporation of BVZ ion pairs in the inner oil phase due to their increase in log P_app_ compared with BVZ.

When BVZ is introduced as ion pair in PL2, the release rate is slower than that obtained when BVZ alone was present, confirming the formation of ion pairs. Moreover, the formation of the BVZ–AOT ion pair and the introduction into the ME modifies the gelation time, decreasing it, thus stabilizing the ME. The release rate of BVZ as ion pairs from PL2 in 24 hours was very low and dependent on log P_app_ value; moreover, in some cases, lag time longer than two hours were observed. For these reasons, BVZ ion pair containing O/W ME seem to be not suitable for the topical corneal administration. On the other hand, these in vitro very slow releases are stimulating to plane further studies to exploit any possible way to the use of the BVZ-ion pair containing O/W ME as sustained (more than one month) BVZ delivery systems. The idea might be to propose them for intravitreal administrations.

BVZ aqueous solution has a limited ability to penetrate corneas with intact epithelium, and preliminary permeation tests through the cornea were required to verify the permeation capacity of BVZ from the liquid systems under study. Moreover, as in tube release studies, BVZ ion pairs showed a very slow release; they were not used in the corneal permeation studies.

The BVZ percentage diffused through the cornea from all the studied systems was, in general, very low; due to its high molecular weight, BVZ hardly permeates the corneal structure, which excludes macromolecules with a radius greater than 10Ǻ. Regarding permeation, water percentage had an effect whereby, increasing water content of the delivery system from the W/O ME to O/W ME (M2 to PL2), the corneal permeation was increased. These results support the advantage of using BVZ containing O/W ME, probably related to their capability to enhance permeation. On the other hand, BVZ released by the liquid crystal system (LC2) accumulates on the cornea more than the other systems under study, proving to be an excellent topical BVZ release system. Presumably, in vivo, M2, upon contact with the tear fluid, could transform into the LC system (LC2) by changing its viscosity and therefore increasing the residence time on the cornea as well.

From the release tests, however, it was possible to evaluate the maintenance of the dose-dependent activity of the BVZ compared to the free BVZ solution on the ARPE-19 cell line.

Viscosity and rheology studies carried out on most ME formulations allowed to reinforce some hypothesis. The increase in viscosity upon water dilution (tear fluid in case of possible topical administration on the cornea) for the W/O ME, or with the temperature increase, typical of the ocular surface, for O/W ME, would allow prolonging in situ permanence of the BVZ release system. Both the proposed systems increase their viscosity, and therefore they could keep BVZ on the cornea, preventing or slowing down the washout due to eye drainage, improving its residence time. In addition, the results of adhesion tests confirmed a good adhesion capacity of the W/O systems. Moreover, MEs can increase the drugs’ retention in the cornea by virtue of their solubilizing power, which allows the partitioning of the dissolved fraction of the drug into the epithelium, in addition to their nanometer size. As LC is a more rigid system than the ME, it passes to a less extent through the cornea, but it remains on the corneal surface and, after 24 h, the LC system allows a high BVZ accumulation, greater than the ME systems.

All the obtained results allowed to do an accurate screening on different ME and LC systems containing BVZ as such or as a hydrophobic ion pair, concluding that the different BVZ location, viscosity and release patterns may be exploited for several purposes and selecting those that can be proposed for topical corneal NV therapy.

## 5. Conclusions

The MEs studied in this research, both W/O and O/W, are able to change their viscosity under conditions that mimic the corneal environment (presence of tear fluid or temperature increase). Moreover, they incorporate BVZ, which is then able to permeate the cornea. A prolonged precorneal residence time could reduce the frequency of administration and promotes the crossing of the various ocular barriers. Therefore these systems can be considered a promising strategy for the treatment of the corneal NV secondary to a variety of the corneal diseases.

## Figures and Tables

**Table 1:** Decrease percentage of monomeric bevacizumab (BVZ) concentration (ΔC%) in normal saline and in a vehicle (an aqueous solution with the same composition of the commercial product).

Initial Concentration	1.25 mg/mL	5 mg/mL	1.25 mg/mL	5 mg/mL
Time (Days)	ΔC% in Normal Saline		ΔC% in a Vehicle	
1	−24	−10	0	0
4	−30	−12	0	0
30	−33	−14	−1.8	−1.2

**Table 2:** Yield of ion-pair formation and log P_app_ ± SD of ion pairs.

Ion Pair	Yield (%)	Log P_app_ ± S.D.
BVZ	-	0.90 ± 0.02
BVZ–AOT (1:75)	90.0 ± 0.8	2.77 ± 0.08
BVZ–AOT (1:150)	91.7 ± 1.0	2.40 ± 0.07
BVZ–CEF (1:150)	76.0 ± 0.4	2.29 ± 0.08

**Table 3:** Composition of the liquid systems formulated without BVZ or with BVZ as Avastin^®^ diluted with a vehicle.

	M1 (mg)	M2 (mg)	LC1 (mg)	LC2 (mg)	PL1 (mg)	PL2 (mg)
Ethyl oleate	313	313	313	313	17	17
Labrasol^®^	-	-	-	-	30	30
Epikuron^®^ 200	220	220	220	227	-	-
Polysorbate 80	244	233	286	250	265	318
Water	150	-	350	170	-	-
Pluronic^®^ F-127 solution	-	-	-	-	937	773
Avastin^®^	-	150 µL ^1^	-	150 µL ^1^	-	80 µL

^1^ Avastin was previously diluted to 8.33 mg/mL of BVZ with a vehicle.

**Table 4:** Viscosity of colloidal systems formulated, measured at 0.08 s^−1.^

Sample	Temperature (°C)	Viscosity × 10^3^ ± S.D. (mPa·s)
M1	25	0.89 ± 0.11
LC1	25	105.58 ± 3.50
PL1	18	1.25 ± 0.10
	32	3604.03 ± 197.28
Pluronic^®^ F127 gel	18	1.20 ± 0.12
	32	3647.22 ± 231.00

**Table 5:** Percentage of BVZ (±S.D.), free and as ion pair, released from Pluronic^®^ F127-containing systems vs. time.

Time (h)	Pluronic^®^ F127 + BVZ at 32 °C	PL2 Free BVZ at 32 °C	PL2 BVZ–AOT (1:150) at 32 °C	PL2 BVZ–CEF (1:150) at 32 °C	PL2 BVZ–AOT (1:150) at 18 °C	PL2 BVZ–CEF (1:150) at 18 °C
		Release (%) ± SD				
0	0	0	0	0	0	0
0.25	12.10 ± 0.51	7.05 ± 0.24	0	0	0	0
0.5	21.85 ± 0.70	14.10 ± 0.46	0	0	0	0
0.75	30.45 ± 1.32	21.25 ± 0.31	0	0	0	0
1	44.40 ± 0.93	30.15 ± 1.68	0	2.03 ± 0.28	0	7.36 ± 0.58
2	51.85 ± 1.70	41.95 ± 1.21	5.15 ± 0.75	14.17 ± 0.90	5.05 ± 0.70	28.83 ± 0.87
4	69.55 ± 1.95	52.85 ± 1.45	7.40 ± 0.76	16.85 ± 1.10	13.75 ± 1.43	45.23 ± 1.93
7	78.62 ± 1.18	70.35 ± 1.48	9.05 ± 0.88	21.65 ± 0.91	19.85 ± 1.27	59.69 ± 1.57
24	91.23 ± 1.81	89.20 ± 1.50	14.50 ± 1.12	25.15 ± 1.43	24.40 ± 1.41	68.50 ± 1.86

**Table 6:** Increase of cornea absorbance (ΔAU) after contact with microemulsion systems and with glutathione bicarbonate Ringer (GBR) buffer.

Samples (Abs 0 Min)	ΔAU 1 Min	ΔAU 10 Min
GBR buffer (0.153)	0.008 ± 0.001	0.009 ± 0.001
M2 (0.171)	0.035 ± 0.011	0.057 ± 0.015
PL2 (0.114)	0.019 ± 0.002	0.021 ± 0.003

**Table 7:** Amount of permeated and accumulated BVZ after 24 h, expressed as a percentage, through the cornea, released from the ME systems under study.

Sample	% Diffused BVZ in 24 h	% Accumulated BVZ in 24 h
BVZ solution (Avastin®)	0.40 ± 0.11	5.16 ± 0.66
M2	0.56 ± 0.09	15.24 ± 0.95
LC2	0.70 ± 0.11	19.89 ± 0.83
PL2	1.56 ± 0.09	4.08 ± 0.37

**Table 8:** Flow rate of water-in-oil (W/O) ME (M2) and LC (LC2) along a hardened gel simulant surface tension of tear fluid.

Sample	Flow Rate (cm·s^−1^)
Distilled water	4.81 ± 0.20
M2	0.08 ± 0.02
LC2	Not Detectable *

* under detection limits.

## Data Availability

Please refer to suggested Data Availability Statements in the section “MDPI Research Data Policies” at https://www.mdpi.com/ethics.

## Supplementary Materials

The following are available online at https://www.mdpi.com/1999-4923/13/2/258/s1, Figure S1: CD absorbance spectrum of BVZ after breaking of ion pair in 190–240 nm region. The gray box is due to the region of the spectrum heavily affected by the presence of electrolytes and, thus rendering the CD signal impossible to analyze; Figure S2: Monomeric BVZ form in the ME systems, confirmed by AF4 analysis (a) in O/W ME (PL2) and (b) in the W/O ME (M2).
