# Supplementary material for: Assessment of In-Situ Gelling Microemulsion Systems upon Temperature and Dilution Condition for Corneal Delivery of Bevacizumab
Source: Pharmaceutics. 2021 Feb 13;13(2):258. doi: 10.3390/pharmaceutics13020258 (PMC7918509; doi:10.3390/pharmaceutics13020258)
Supplement: Supplementary file 1 [file pharmaceutics-13-00258-s001.pdf]

pharmaceutics

MDPI

Supplementary Materials: Assessment of in Situ-Gelling Microemulsion Systems Upon Temperature and Dilution Condition for Corneal Delivery of Bevacizumab

Elena Peira, Giulia Chindamo, Daniela Chirio, Simona Sapino, Simonetta Oliaro-Bosso, Gianmario Martra, Erica Rebba, Pavlo Ivanchenko and Marina Gallarate

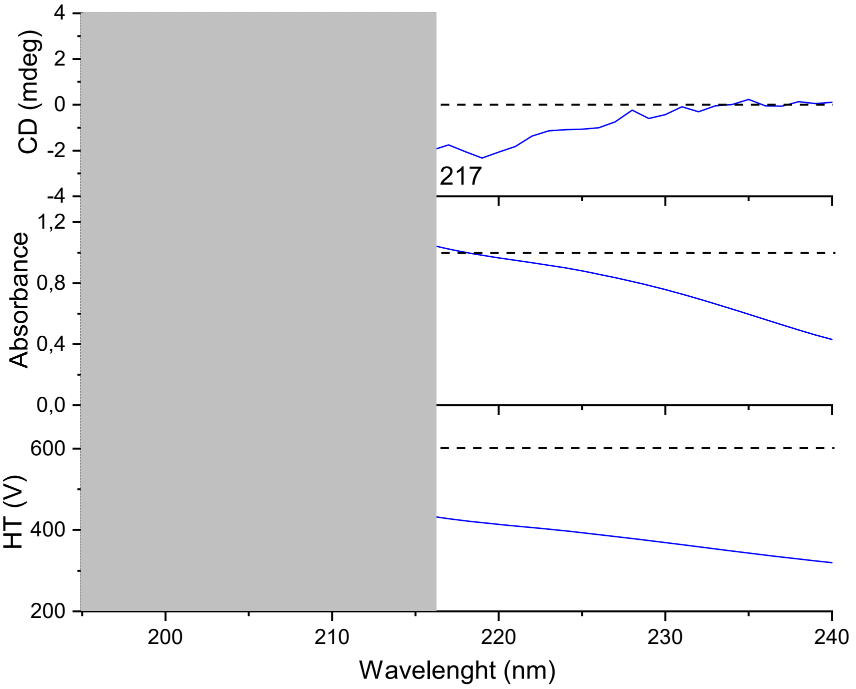

Figure S1. Monomeric BVZ form in ME systems, confirmed by AF4 analysis. a. in O/W ME (M2) and b. in W/O ME (PL2).

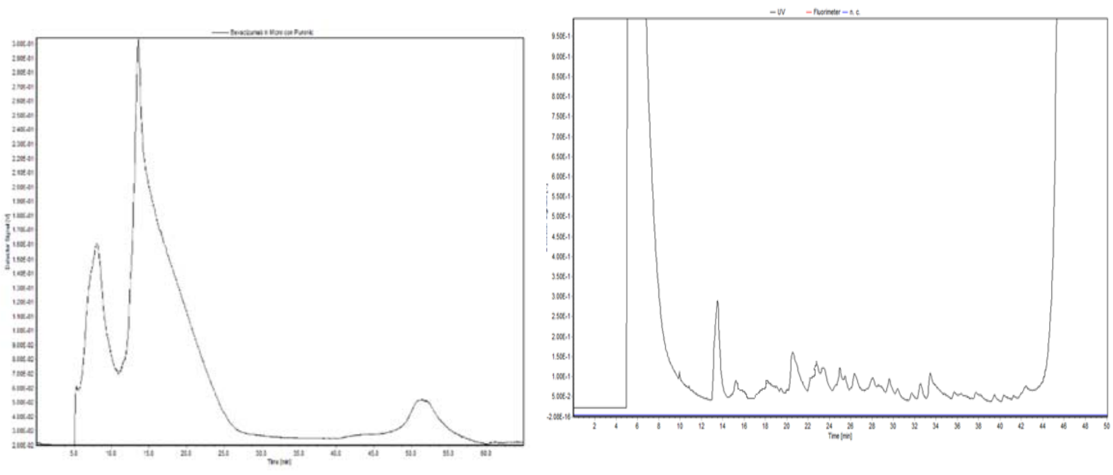

(**a**) (**b**)

Figure S2. Monomeric BVZ form in ME systems, confirmed by AF4 analysis. (a). in O/W ME (PL2) and b. in W/O ME (M2).
